# Three Cases of Abdominal Aneurysm

**Published:** 1907-08-10

**Authors:** 


### Clinical Points.

### THREE CASES OF ABDOMINAL ANEURYSM.

Abdominal aneurysm is by no means a common condition, at any rate in the South of Britain. Aneurysm of the aorta is much less rare in the North, where arduous work in collieries, iron foundries and so forth exceeds the generality of physical exertion elsewhere, whilst syphilis and drink, the predisposing factors to aneurysm, are probably as prevalent there as in other places. One is naturally chary of diagnosing that which is rare; consequently abdominal aneurysm is sometimes overlooked. The following cases may therefore be of interest; in one of them the diagnosis was correctly made, in the other two not so.

Case 1.—*Aneurysm of the abdominal aorta; erosion of the spine; compression of the cord; paraplegia.*

A man aged 30, a sailor and a free drinker had syphilis five years before his present illness started. The hard chancre was followed in five weeks by a secondary rash on the legs, chest, and back. This went away without any serious treatment; the attack of syphilis was regarded as quite mild. Five months before his death he began to have pains across his loins and in his right flank, accompanied by a feeling of weakness in the legs. The pain in the loins increased, and extended round the abdomen as if a cord were tied round the body. The weakness in the legs increased so much that he had to stay in bed; the legs were moderately spastic, not wasted; weak and numb, though not absolutely anæsthetic; the knee jerks were exaggerated, the plantar reflexes were extensor, and there was ankle clonus on both sides. There was no incontinence of fæces, the bowels being confined and acting only once in about five days; he had occasional difficulty in passing urine, though there was no stricture; this difficulty never amounted to retention. His features expressed suffering. Examination of the spine revealed neither deformity nor local tenderness. The heart, lungs and urine were normal; abdominal palpation discovered no lump; the nervous system was normal, except for the trouble in the legs.

The diagnosis was transverse myelitis, which was attributed to the syphilis; but on looking back at the case now, it is quite clear that one point on which much greater stress should have been laid in diagnosing the cause of the myelitis was the presence of so much pain. Transverse myelitis is of two main kinds, namely:—

I. *Primary*, when the whole of the trouble is within the meninges; in which case there is no pain.

II. *Secondary*, due to compression of the cord from without by such things as tuberculous spinal caries, exudation or callus after injury to the vertebræ, secondary deposits of new growth, or aneurysm eroding the vertebræ; in all these cases the posterior nerve roots become involved before the cord itself becomes compressed.

Interference with the cord alone causes no pain, but irritation of posterior nerve roots gives rise to much; hence the one point upon which most stress should be laid in distinguishing between primary and compression myelitis is the presence or absence of pain. Had more attention been paid to this in the above case, compression myelitis would have been diagnosed rather than primary syphilitic myelitis; but even then it is more than likely that tuberculous spinal caries would have seemed more probable than aneurysm, for two reasons: first, that there was no tumour to be felt, still less a pulsatile tumour; secondly, that it is distinctly unusual for an abdominal aneurysm to erode the vertebræ so far as to lead to compression of the cord. There was no history of injury, and no evidence of new growth as a source for secondary deposits, so that these would both have been put out of court; the main arguments against spinal caries were the general healthy appearance of the patient, and the facts that he had followed an outdoor life upon the sea, and was older than the usual age for spinal caries. In another similar case of compression paraplegia, therefore, the diagnosis of abdominal aneurysm may be arrived at by a process of exclusion, when the patient is a vigorous man of 30, has girdle pain, has suffered from insufficiently treated syphilis, is a hard drinker, and has been engaged in an arduous occupation.

A week after the diagnosis of syphilitic myelitis and paraplegia made, the patient having then been confined to bed for three months though only lately under observation, he suddenly became prostrate and blanched. It was clear that he had had a sudden internal hemorrhage; rupture of an aneurysm was then suspected, and this was confirmed by autopsy next day. The aorta was extensively affected by syphilitic atheroma; opposite the first lumbar vertebræ there was a saccular aneurysm the size of half an orange; this had eroded the bodies of the vertebræ and compressed the lower part of the cord; the intervertebral discs were intact as usual; there was a rupture in the right side of the sac, and from this about a pound and a half of blood had escaped into the retroperitoneal tissues, filling the right flank and right iliac fossa. The reason why the aneurysm could not be palpated during life was that it arose quite posteriorly; the aorta itself lay in front of the sac. If the tumour extend in front of the aorta the difficulty in diagnosis by palpation is difficult enough, but it is much more so when a small aneurysmal sac is placed between the aorta and the body of the vertebræ.

Case 2.—*Multiple aneurysms of the aorta; symptoms resembling those of renal colic; death by rupture.*

A man aged 49, a shoemaker by trade, and of professedly temperate habits for many years, had untreated syphilis when a young man, though no secondary rash followed the chancre. He was now complaining of severe pain covering an area the size of a shilling behind the region of his left kidney; the pain came on gradually, but increased in severity, extending by degrees to the front of the abdomen, and for two or three days latterly it shot down into the left testicle. It was so severe that he was quite unable to lie upon his back, and he was in agony when he moved or when his back was touched. The pain was along the course of the left genito-crural nerve to the front of the thigh and to the testicle. The heart, lungs, and general nervous system seemed natural; nothing abnormal was detected on examination of the abdomen. The urine was passed with slight difficulty but without pain; it measured 40oz. per diem, was clear when passed, depositing urates on standing, and microscopical examination revealed neither pus, blood corpuscles, nor excess of crystals. The $x$-rays gave no evidence of stone, but all the symptoms seemed to point to renal colic.

At the autopsy the aorta was found extensively diseased; there was a small thoracic aneurysm to the right of the trachea, partially compressing this, but causing neither symptoms nor signs during life—the kind of lesion which, by rupture, sometimes leads to sudden unexpected death. There was a second small aneurysm in the descending thoracic aorta; and immediately below the diaphragm was a third aneurysm, the one that had caused all the symptoms. Its orifice was one and a-half inches long and three-quarters of an inch wide; it extended back to the bodies of the vertebræ, but eroded them only a little; the main body of the sac extended to the left behind the left kidney, which was raised in front of it. The lumbar plexus was stretched across the sac, whence arose the extreme pain first in the genito-crural nerve, resembling renal colic, and later in the external cutaneous. The sac had burst downwards, and there was much retro-peritoneal blood clot reaching as low as Poupart's ligament.

The explanation of the symptoms in this case is easy enough after the event; renal colic was so closely simulated that it is likely that this would be the diagnosis again in a similar case; on the other hand, when the pains had extended beyond the distribution of the genito-crural nerve to that of the external cutaneous, renal colic no longer fitted in well. The exact distribution of the pains in such a case is very important; and another point upon which stress might well be laid is the fact that the pain was almost continuous, even when the patient was in bed, and worse at night than in the day; whereas the pain of renal calculus is intermittent, and nearly always disappears when the patient lies still in bed. The pain of renal growth or of tuberculous kidney may persist in spite of rest, and these were possibilities in the case; though in them the pain is mainly in the loin, seldom radiating into the groin and testicle. The fact that neither blood, pus, nor crystals were found in the urine was certainly against a renal lesion, but it is never wise to lay too much stress upon such negative evidence; even when much renal mischief is present the urine may appear practically normal sometimes.

Case 3.—*Aneurysm of the abdominal aorta at the cœliac axis; gastric symptoms and pain in the testicle; later, pulsatile tumour felt; rupture behind peritoneum.*

A man aged 34 had syphilis seven years previously, and three months before his death began to have severe abdominal pain, which started immediately after lifting a heavy weight. The pain was chiefly in the epigastric region, and it was soon associated with gastric symptoms, everything being rejected from the stomach. There was a suspicion of gastric ulcer, or of gastric carcinoma at first; soon the diagnosis became obvious; for not only was there a subjective sense of throbbing, and an extension of the pain through to the back in the dorso-lumbar region, but also a pulsatile tumour as big as an orange became palpable in the epigastrium. One curious point was that the patient also complained of great pain in the right testicle, though the testicle itself and the urine seemed normal. No murmur could be heard over the tumour—indeed, the presence or absence of a murmur is of extremely little value in either diagnosing or excluding aneurysm. The autopsy showed a very atheromatous aorta, upon which, at the level of the cœliac axis, was an aneurysm the size of a fist upon the front of the aorta, the superior mesenteric artery arising from it. The solar plexus was stretched over the wall of the sac, accounting for the incessant vomiting. The right genito-crural nerve was also involved, accounting for the pain in the testicle.

The part of the abdominal aorta that is most frequently affected with aneurysm is the upper part, either at the cœliac axis or at the origin of the superior-mesenteric artery. In nearly every case there has been preceding syphilis, the most potent predisposing factor, added to which there have usually been extensive use of alcoholic liquids and the performance of intermittent hard manual work. The aorta may be atheromatous throughout, but very often this cannot be determined, for the rest of the cardio-vascular system may appear normal, and there may be no obvious disease of any of the organs.

The age at which this form of arterial disease is most liable to develop is during the prime of life, between 30 and 50, and more particularly between 30 and 40, when carcinoma is uncommon. Men are much more prone to the disease than are women.

It is not usual for many months to elapse after the formation of a pulsatile abdominal aneurysmal tumour before the disease terminates in death; in the great majority the end is by rupture of the sac behind the peritoneum.

The absence of cachexia and of general disturbance to the system is very observable in aneurysmal disease; the countenance is expressive of pain, but it is without the characteristic appearance of ulceration or of cancer. There may be vomiting from interference with the solar plexus, but as a rule there is no disturbance in the functions of the abdominal viscera; there are no indications of jaundice, nor of ascites, nor of distended veins on the surface of the abdomen; there may be severe pains in the loins over the kidneys, and pain in the testicles, but there is no abnormality in the urine; there is intense abdominal pain, but no evidence of peritonitis. It is by a process of exclusion that the diagnosis must be made when there is no pulsatile tumour to make it obvious. Intense pain in the back or abdomen of a fixed but paroxysmal character in men during ripe manhood, without evidence of functional disturbance of the viscera, is in itself always suspicious of aneurysmal disease.

